# Automatic motion probe setting assist system for cardiac magnetic resonance imaging

**DOI:** 10.1186/1532-429X-15-S1-E21

**Published:** 2013-01-30

**Authors:** Shigehide Kuhara, Shuhei Nitta, Taichiro Shiodera, Tomoyuki Takeguchi, Kenichi Yokoyama, Rieko Ishimura, Toshiaki Nitatori

**Affiliations:** 1 MRI Systems Development Department, Toshiba Medical Systems Corporation, Otawara-shi, Tochigi, Japan; 2 Corporate Research & Development Center,Toshiba Corporation, Kawasaki-shi, Japan; 3 Department of Radiology, Faculty of Medicine, Kyorin University, Mitaka-shi, Japan

## Background

Planning assist systems for cardiac MR examinations are necessary for easier operation and shorter examination times. This is also true for motion probe setting in whole-heart MR imaging. In previous reports, we proposed an automatic planning assist system for couch adjustment, local shimming, and axial multislice imaging using single scout volume data of the chest. This system employs an atlas-based segmentation technique, so it can detect not only the heart region but also various anatomical structures in the chest. In the present study, this technique was employed to detect the position of the top of the right hemidiaphragm for motion probe setting. The results were also compared against the degree of interobserver error in manual annotation.

**Figure 1:**
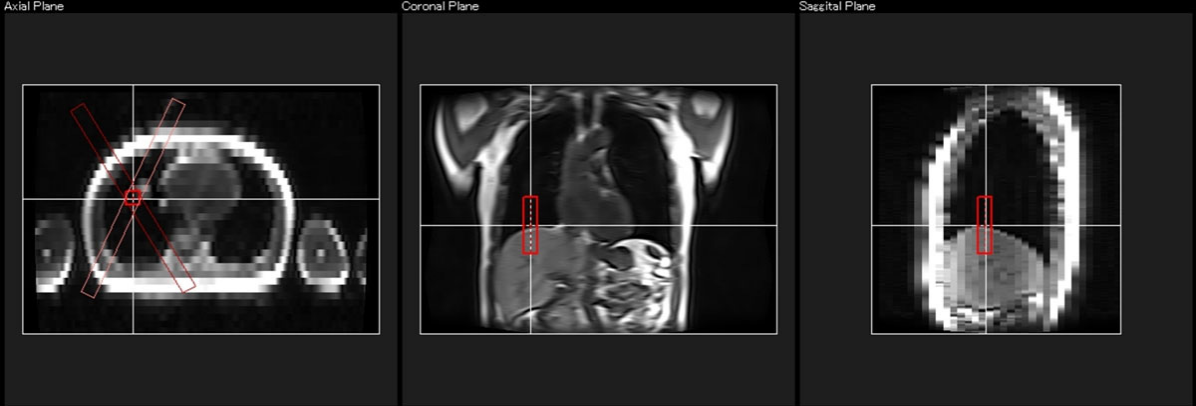
Automatic motion probe setting assist system for MRI exams

## Methods

An ECG-non-gated 3D fast field echo (FFE) single volume covering the entire chest area was acquired using a 1.5-T MRI scanner (Excelart VantageTM powered by Atlas, Toshiba Medical Systems) during a single breath-hold with TR/TE = 3.7/1.3, FOV = 500x350x350 mm3 (coronal slab), and readout/phase/slice encode steps = 256/64/35 in an acquisition time of approximately 9 seconds. The acquired volume was then transformed to match a prepared model volume with manual annotation of the heart region and the position of the top of the right hemidiaphragm, permitting the motion probe region of the input data to be located. Accuracy was assessed by measuring the Euclidean distance between the position of the top of the right hemidiaphragm obtained by our method and that obtained by manual annotation, and evaluation was performed by comparison with the differences between two manual annotations as a measure of interobserver error.

## Results

The proposed method successfully segmented the heart region and detected the position of the top of the right hemidiaphragm for motion probe setting in 48 datasets from 15 healthy volunteers. The processing time was approximately 1.6 seconds (2.5 GHz CPU, single-thread processing). The interobserver error was also measured for 15 datasets from 15 healthy volunteers manually annotated by two operators. The average Euclidean distance error and the interobserver error were 11.04±4.73 and 14.76±19.13 mm, respectively.

## Conclusions

Our previously proposed automatic planning assist system has been extended to motion probe setting. The results of the present study showed that the position of the top of the right hemidiaphragm could be detected by our method almost as accurately as by manual annotation. It is expected that this method should be useful in the clinical setting.

## Funding

No funding was received for this research.

